# Untargeted NMR Spectroscopic Analysis of the Metabolic Variety of New Apple Cultivars

**DOI:** 10.3390/metabo6030029

**Published:** 2016-09-19

**Authors:** Philipp Eisenmann, Mona Ehlers, Christoph H. Weinert, Pavleta Tzvetkova, Mara Silber, Manuela J. Rist, Burkhard Luy, Claudia Muhle-Goll

**Affiliations:** 1 Institute of Organic Chemistry, Karlsruhe Institute of Technology, Fritz-Haber-Weg 6, 76131 Karlsruhe, Germany; philipp-michael.eisenmann@kit.edu (P.E.); pavleta.tzvetkova@kit.edu (P.T.); mara.silber@student.kit.edu (M.S.); burkhard.luy@kit.edu (B.L.); 2 Department of Safety and Quality of Fruit and Vegetables, Max Rubner-Institut, Haid-und-Neu-Straße 9, 76131 Karlsruhe, Germany; ehlers.mona@web.de (M.E.); christoph.weinert@mri.bund.de (C.H.W.); 3 Institute for Biological Interfaces 4, Karlsruhe Institute of Technology, P.O. Box 3640, 76021 Karlsruhe, Germany; 4 Department of Physiology and Biochemistry of Nutrition, Max Rubner-Institut, Haid-und-Neu-Straße 9, 76131 Karlsruhe, Germany; manuela.rist@mri.bund.de

**Keywords:** metabolomics, multivariate analysis, quality control, malus domestica

## Abstract

Metabolome analyses by NMR spectroscopy can be used in quality control by generating unique fingerprints of different species. Hundreds of components and their variation between different samples can be analyzed in a few minutes/hours with high accuracy and low cost of sample preparation. Here, apple peel and pulp extracts of a variety of apple cultivars were studied to assess their suitability to discriminate between the different varieties. The cultivars comprised mainly newly bred varieties or ones that were brought onto the market in recent years. Multivariate analyses of peel and pulp extracts were able to unambiguously identify all cultivars, with peel extracts showing a higher discriminative power. The latter was increased if the highly concentrated sugar metabolites were omitted from the analysis. Whereas sugar concentrations lay within a narrow range, polyphenols, discussed as potential health promoting substances, and acids varied remarkably between the cultivars.

## 1. Introduction

Apples are among the most cultivated fruit crops and their metabolic profile has already been extensively characterized. The metabolome, the sum of all metabolites, varies with many factors like the genetic species, environment, or disease. Metabolomic studies on crops often address questions like species identification in quality control [1], influence of cultivation [2], or storage conditions [3]. Untargeted approaches using mass spectrometry or NMR techniques detect and quantify several substance classes simultaneously. High resolution NMR spectroscopy shows resonances of all soluble compounds exceeding a concentration threshold of approximately 5–10 µM.

Most studies on apples published so far were targeted analyses that focused on specific metabolite classes with substance specific analytical techniques [2,4,5]. Untargeted analysis methods giving a more comprehensive view on the metabolome have also been applied but to a lesser extent. Tomita et al. [6] compared pulp and juice of five established commercial cultivars grown in Japan or New Zealand by high resolution liquid state NMR spectroscopy. They could clearly distinguish the different geographic origins by multistep principal component analysis (PCA). Vermathen and coworkers assessed the suitability of high resolution magic angle spinning NMR spectroscopy to distinguish apple tissue derived from three common cultivars: Braeburn, Golden Delicious, and Rubens [7,8]. Cuthbertson et al. [9] employed gas chromatography coupled to mass spectrometry of pulp and peel to distinguish six cultivars (Golden Delicious, Red Delicious, Gala, Fuji, and Granny Smith). They performed PCA, cluster analysis, and correlation analysis to identify metabolites specific for certain traits such as antioxidant activity, total phenolic compounds, and total anthocyanins. De Paepe et al. performed an LC–MS study that focused on the analyses of known phenolic compounds [10]. Forty-seven cultivars belonging to classic/new, heritage, or red-flesh cultivars were analyzed and could be unambiguously identified in PCA. Furthermore, heritage apples that were mainly grown under organic agricultural practice showed a remarkably distinct phenolic pattern. LC–MS was also used for authenticity measurement of fruit juice [11]. Farneti et al. investigated the volatile organic compounds of a large variety (190) of old and new cultivars by proton transfer reaction time-of-flight mass spectrometry, on the basis of which they separated the cultivars in two major groups, characterized by their content in esters and alcohols [12].

Modern apples have been developed from a handful of ancestors that are quite susceptible to a number of fungal and bacterial diseases. To minimize the use of chemicals and employ, for example, organic farming, breeding programs focus their research activities on the development of resistance against common apple diseases. Between 1984 and 2004 more than 1000 new cultivars have been registered for Europe [13]. The examples given above show that analysis of the metabolome allows identification of different species by providing unique fingerprints. Thus, analyses of the apple fruit metabolome may assist in the selection of desired traits. In apples these can be a high content in polyphenolic compounds discussed as antioxidative agents [14], or resistance against prevalent apple crop pests like scab or mildew caused by *Venturia inaequalis* and *Podosphaera leucotricha*, respectively. Sciubba et al. recently correlated higher concentrations of known antifungal activity conferring metabolites with scab resistance, but the study comprised only two cultivars [15].

Here, we have performed an untargeted NMR-based metabolome analysis of 14 apple cultivars harvested in 2014 (Table 1). The cultivars analyzed in this screening study were selected from an on-farm evaluation program aiming to compare the basic agronomic characteristics (especially time of harvest, yield, and average fruit weight) of the newly bred cultivars with established reference cultivars like Elstar in a pesticide-reduced growing system. These cultivars were already selected from a larger range of cultivars because they all proved to have favorable properties concerning cultivation and consumer expectations beforehand. More precisely, they all were predominantly red-skinned because this meets consumer expectations best, at least in Germany. Likewise, all of these cultivars were *a priori* known to have an attractive sensory profile. Further, most of the cultivars were resistant against scab and mildew and also did not show any symptoms of other diseases at the point of harvest. We deliberately selected phenotypically rather similar cultivars because they all had—from the growers’ and the consumers’ perspective—favorable properties and have therefore been introduced into the market recently or have a potential to appear on the market within the next years. Both pulp and peel extracts were analyzed to look for specific marker patterns that characterize the individual cultivars.

## 2. Results

### 2.1. Metabolite Extraction and Identification

The chemical shifts of several metabolites occurring in apple are highly influenced by the pH value of the buffer. Even small changes can shift the resonances of compounds with solvent exchanging hydrogens, mainly organic acids. This affects the efficiency of automated bucketing and thus the feasibility of NMR-monitored quality control is highly dependent on a tight control of the pH. To aggravate the problem, ^1^H NMR spectroscopy requires that the buffer contains no or as few as possible hydrogens that would otherwise dominate the spectrum. This excludes many common biological buffers like HEPES or TRIS-HCL. Finally, the buffer concentration should be moderate, as high salt concentrations impair the required high homogeneity of the magnetic field. For these reasons, we chose 200 mM phosphate buffer, pH 3.04 to extract apple pulp and 200 mM deuterated acetate buffer, pH 4.08 for peel extracts. Although this buffer choice drew near to the natural pH of apples, it was not fully sufficient as apples are rich in organic acids. After extraction, the pH of pulp extracts varied between 2.7 and 3.3 and that of peel extracts between 3.8 and 4.3. This variability did not affect the spectra of pulp extracts, however the aromatic region (Figure S1) in peel extracts showed a certain degree of resonance shifts due to small pH changes. To avoid problems with the statistical analysis, larger bucket sizes were chosen for these areas.

Ten different samples for each cultivar were collected to assess the variability within a cultivar, where each sample combined material from five fruits each. This approach was chosen to reduce obvious effects stemming, e.g., from different exposures to sunlight.

**Figure 1:**
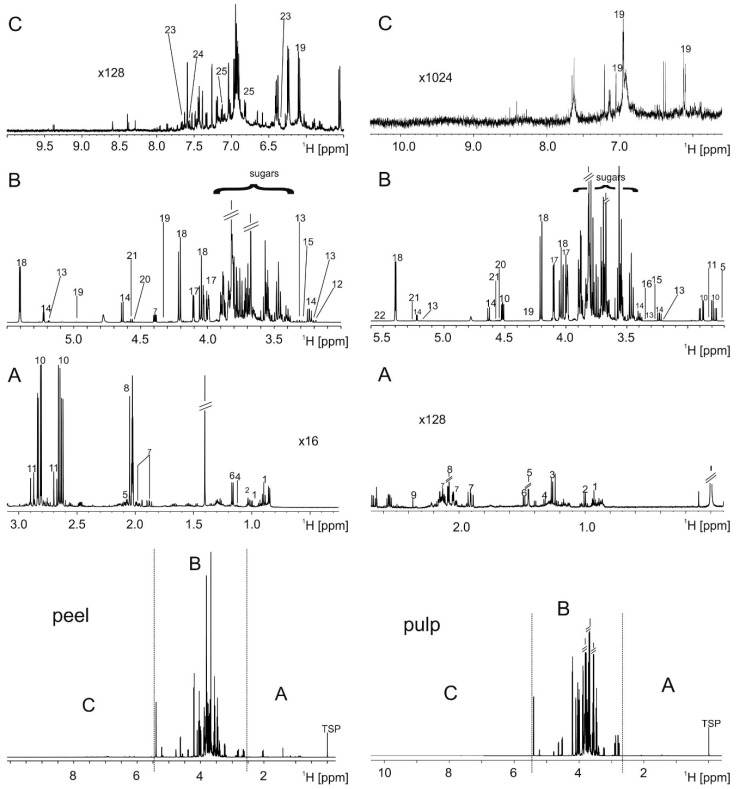
^1^H-NMR spectra of peel (**left**) and pulp (**right**). The bottom panels show the entire spectra. Regions that are magnified in (A–C) are indicated. (A) Amino acid region; (B) sugar region; and (C) aromatic region. Metabolites: 1 isoleucine, 2 valine, 3 rhamnitol, 4 lactate, 5 citramalic acid, 6 alanine, 7 quinic acid, 8 acetate, 9 pyruvate, 10 malate, 11 citrate, 12 choline, 13 xylose, 14 glucose, 15 myo-inositol, 16 betaine, 17 fructose, 18 sucrose, 19 epicatechin, 20 tartrate, 21 galactose, 22 arabinose, 23 chlorogenic acid, 24 p-coumaric acid derivative, 25 phlorizin.

Figure S2 shows that pulp extracts were highly comparable within the 10 samples. Peel extracts showed the abovementioned slight variations in peak position in some areas due to minor pH variances observed. Spectra of pulp and peel extracts were dominated by sugars resonances (glucose, sucrose, fructose), which comprised 96%–98% of the intensity in pulp extracts and still >94% in peel (Figure 1). The second highest concentration was found for the organic acids malate and citrate, which ranged between 2% and 4%. All other components had markedly lower intensities. Peak assignment was achieved from databases and spiking (Figure S4). Spectra of pulp and peel extracts showed a predominantly similar composition for metabolites resonating between 5.5 and 0.8 ppm like sugars and aliphatic compounds (e.g., the amino acids), yet a few resonances were found only in respective subsets of cultivars (Figure S4).

Pulp and peel extracts differed, however, considerably in the aromatic area from 6.5 to 9 ppm, where resonances of polyphenolic compounds are found. These were low in fruit extracts, but enriched in peel extracts, where they comprised up to 1% of the total intensity. Strikingly, the NMR study of Tomita et al. [6] on juice or fruit extracts showed a considerably lower amount of polyphenolic compounds. Whether this is an inherent feature of the cultivars investigated or due to cultivation conditions or year of growth cannot be answered on the basis of our data.

The entire bucket list comprised 116 buckets. Twenty-five compounds were identified (Figure 1, Table S1), of which many have several resonances. Thus approximately 42% of all resonances in the spectra could be assigned.

### 2.2. Multivariate Analysis to Differentiate Cultivars

**Figure 2:**
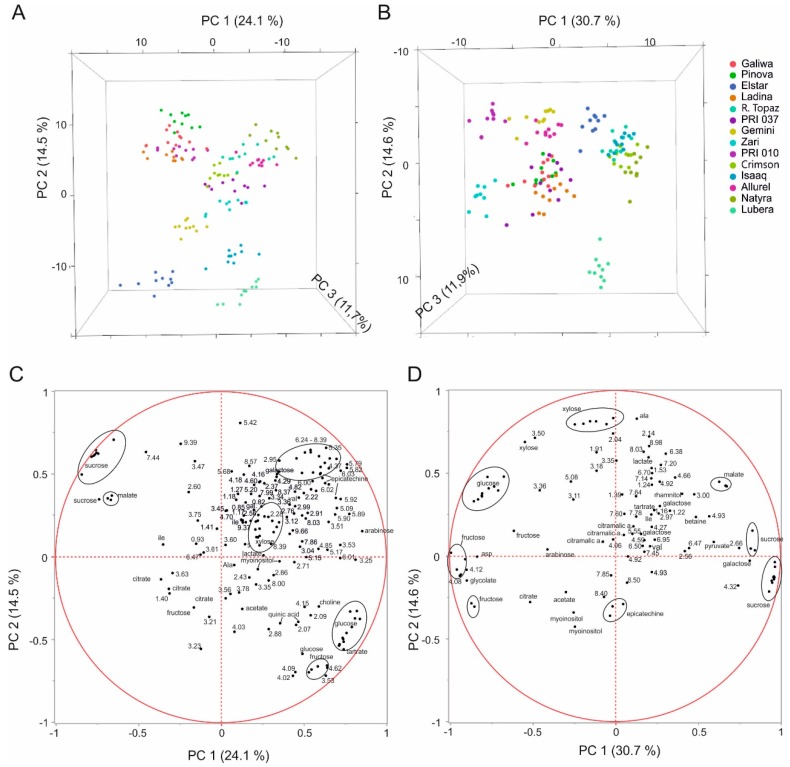
Principal component analysis (PCA) of peel (**A**) and pulp (**B**). (**A,B**) Scores plot showing three PCs (principal component) with their respective variation. Peel: R^2^X (PC1) = 24.1%, R^2^X (PC2) = 14.5%, R^2^X (PC3) = 11.7%, Q^2^ (PC1) = 21.4%, Q^2^ (PC2) = 15.3, Q^2^ (PC3) = 1.7. Pulp: R^2^X (PC1) = 30.7%, R^2^X (PC2) = 14.6%, R^2^X (PC3) = 11.9%, Q^2^ (PC1) = 28.9%, Q^2^ (PC2) = 12.1, Q^2^ (PC3) = 11.0. (**C,D**) Corresponding loadings plot. Loadings are labeled with substance name or ppm value. In case of overlapped resonances, only the ppm value is given.

We performed principal component analyses to assess whether the metabolite spectrum can be used as criterion to identify the different cultivars (Figure 2, Figure S5). For pulp and peel extracts, the first three principal components representing 52.3% (peel) and 57.2% (pulp) of the total variance, were sufficient to distinguish between most of the 14 cultivars (Figure 2A,B). Within each cultivar group, samples were quite dispersed and some groups overlapped at the border (e.g., Red Topaz, Crimson Crisp, Natyra), so that they could only be separated by taking further principal components into consideration. Peel extracts yielded a better separation with more distinct grouping and a lower number of PCs (up to PC5) were needed for unambiguous identification than for pulp (up to PC6).

The loadings plot highlights the metabolites responsible for the separation of the different cultivars (Figure 2C,D). PC1 of pulp and peel extracts was dominated by the sugar compounds where sucrose and malate were negatively correlated with glucose and fructose. PC2 was influenced differently in pulp and peel. In pulp, xylose was negatively correlated myo-inositol and epicatechin. In peel, polyphenolic compounds (ppm values > 6.2 ppm) also contributed to the distinction of the varieties.

The 14 quality control samples generated from a pool of all cultivars lay at the coordinate origin in a narrow area indicative of the high reproducibility of the method (Figure S6). Standard deviations for well-resolved compounds in pulp extracts lay in the range of 0.4% for medium to highly concentrated compounds like glucose, malate or citrate.

Less concentrated metabolites like rhamnitol or galactose had standard deviations around 3%–4%. A similar value was also achieved for myo-inositol that is partially overlapping with glucose. Even compounds like pyruvate or epicatechin, that were barely above noise level could be quantified but with a comparably high standard deviation of 28% or 27%, respectively.

**Figure 3:**
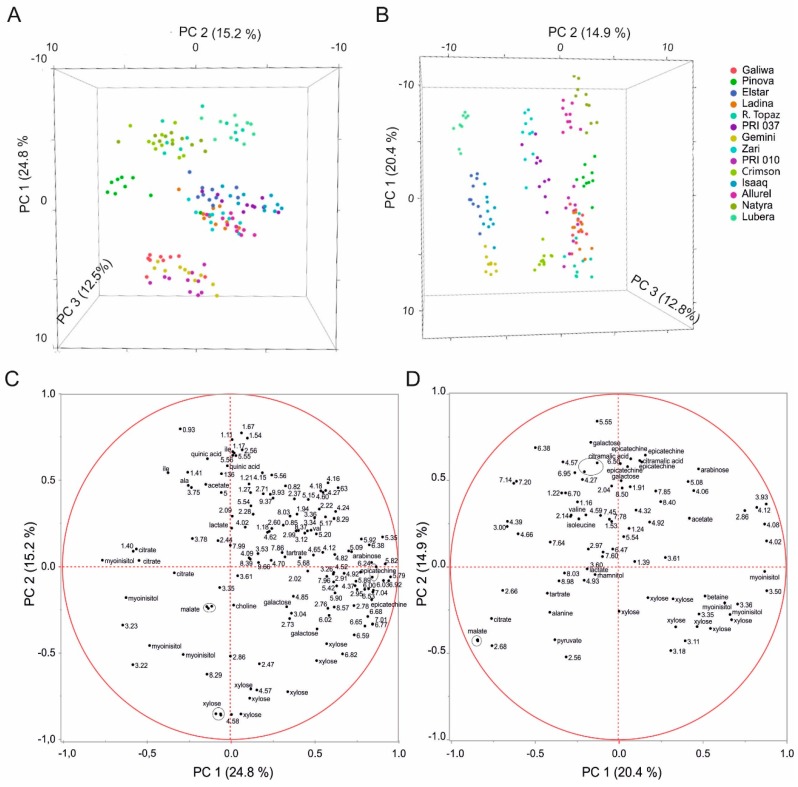
PCA of peel (**A**) and pulp (**B**) omitting sugars from the analyses. (**A,B**) Scores plot showing three PCs (principal component) with their respective variation. Peel: R^2^X (PC1) = 24.8%, R^2^X (PC2) = 15.2%, R^2^X (PC3) = 12.5%, Q^2^ (PC1) = 22.5%, Q^2^ (PC2) = 12.7, Q^2^ (PC3) = 12.1. Pulp: R^2^X (PC1) = 20.4%, R^2^X (PC2) = 14.9%, R^2^X (PC3) = 12.8%, Q^2^ (PC1) = 16.1%, Q^2^ (PC2) = 11.1, Q^2^ (PC3) = 9.8. (**C,D**) Corresponding loadings plot. Loadings are labeled with substance name or ppm value. In case of overlapped resonances, only the ppm value is given.

We further performed a PCA omitting the sugars signals dominating the spectrum to quantify the contributions of lower concentrated metabolites (Figure 3). In this way a more distinct grouping was achieved with fewer PCs. The effect was more pronounced for peel where only three PCs (representing 52.5% of the variance) were sufficient to achieve unambiguous classification of each cultivar, whereas three PCs (representing 47.6% of the variance) were still not fully sufficient for pulp. The content in polyphenolic compounds was now a decisive criterion. High epicatechin and other phenolic compounds contributed together with low citrate and myo-inositol levels to PC1 in peel. PC2 was dominated by high quinic acid, alanine, and isoleucine levels and low levels of xylose, galactose, and lipids (Figure 3C). In pulp, high epicatechin, citramalic acid, and galactose went together with low myo-inositol and citrate levels for PC1, whereas PC2 was mainly determined by high citramalic acid, epicatechin, and galactose concentrations (Figure 3D).

### 2.3. Cluster Analysis

**Figure 4:**
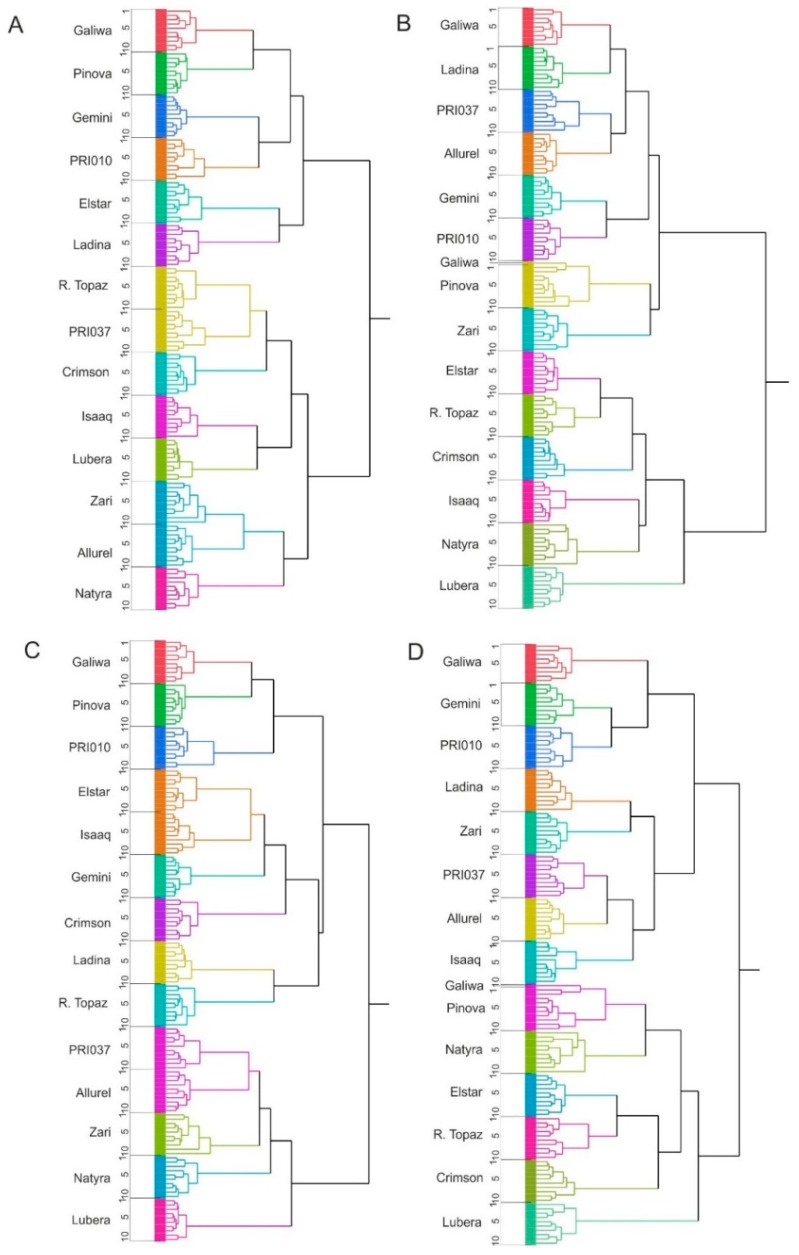
Hierarchical clustering of peel (**A**,**C**) and pulp (**B**,**D**) extracts. (**A**,**B**) All metabolites were used for analysis; (**C**,**D**) sugars were omitted from the analysis.

The high number of cultivars precludes an easy analysis of the relationships between the cultivars. Therefore, we performed a hierarchical cluster analysis (Figure 4). All samples from a given cultivar clustered together regardless of whether sugars were included or omitted from the analysis. The only exception was one sample of Galiwa that clustered together with Pinova Evelina, but the reason for that was not clear. Omitting the sugars, however, changed the relationship between the cultivars. Cultivars that are related like red Topaz and Ladina (parent and offspring) or Elstar v.d. Zalm and Zari (parent and offspring) or Allurel and Natyra (the latter share a common parent: Elise) did not necessarily cluster together.

We also performed partial least squares (PLS)/canonical analysis (CA), separating the cultivars into scab mildew resistant (SMR) and non-SMR. Although the groups could be separated, the variation in concentration for those metabolites, that were supposed to be responsible for the separation, was not significant. As a PLS–CA with randomized assignment of the cultivars to either of the groups achieved a seemingly as good separation, we concluded that the fruit metabolome variation within the current set of cultivars does not allow for the investigation of disease susceptibility.

## 3. Discussion

Since modern apples have been developed from a handful of ancestors [16,17], it was not to be expected *a priori* that their fruit metabolomes would be sufficient for discrimination between the sometimes closely related cultivars of this study. Of the 14 cultivars used in this study at least 6 descend from Golden Delicious either as direct offspring (Pinova Evelina, Crimson Crisp, and Elstar) or one breeding generation later (Zari, Galiwa, Gemini). Yet, all cultivars could be identified by untargeted analyses both in PCA and in hierarchical cluster analyses. Previous NMR analyses had already shown similar results, but had been performed with lower numbers of and/or a more diverse relationship between the cultivars [6,7,8]. In comparison, the discriminative power of peel extracts was substantially higher than that of pulp extracts. This was mainly due to the presence of aromatic and polyphenolic metabolites that are enriched in peel, but are only present to a low extent in pulp. Omitting highly concentrated sugars resonances from the analysis helped in the discrimination of the cultivars.

**Figure 5:**
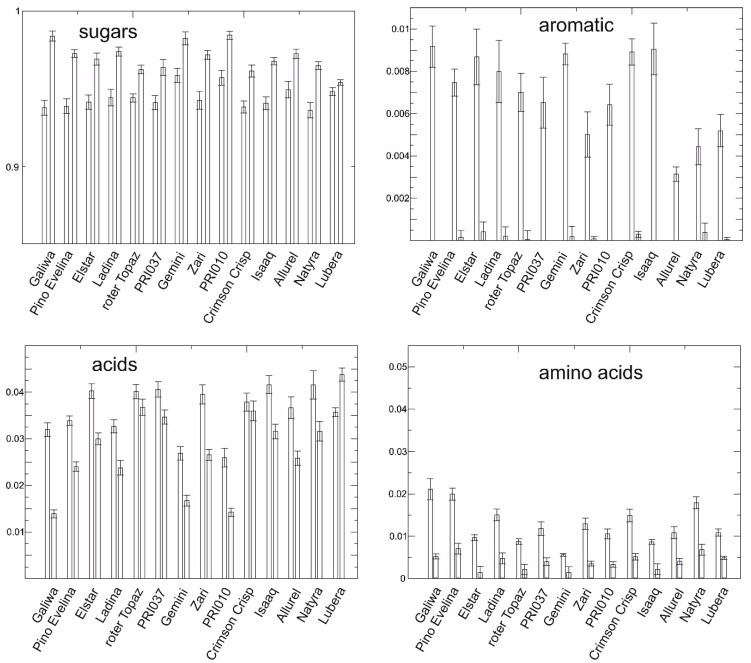
Relative intensities compound classes for all cultivars. Mean intensities and their standard deviation are shown for peel on the left and for pulp on the right where available.

Intensities observed in the NMR spectra directly correlate with relative concentrations of substances and thus can be used for analysis of key compounds. Figure 5 shows an analysis of the total content in metabolite classes, aromatics, sugars, acids and amino acids. Aromatic compounds comprised the region between 6 and 10 ppm, where the resonances of polyphenols, but also compounds with single aromatic rings like chlorogenic acid and coumaric acid or their derivatives occur. Sugars were taken from the intensities of the area between 3.2 and 5.5 ppm, where mainly glucose, fructose, and sucrose resonances are found. The term acids in Figure 5 comprises citrate and malate, and the term amino acids summarizes the resonances between 0.8 and 1.5 ppm. The intensities determined in this way can only be an indication for the respective concentrations as neither the number of compounds in the spectral region nor the number hydrogens giving rise to the signals was taken into account. Figure 5 reveals that the sugar content was quite comparable over all 14 cultivars, probably reflecting the selection criteria for newly bred cultivars with good market potential. Acids and aromatic compounds of peel extracts, however, were more diverse among the cultivars.

Cuthbertson et al. were able to cluster Red Delicious, Golden Delicious, Cox’s Orange Pippin, Gala, Fuji, and Granny Smith according to their genealogy based on the fruit metabolome [9]. The set of cultivars presented here comprised several pairs of closely related cultivars and thus was well-suited to test the discriminative power of the fruit metabolome in terms of genetic relationship. Hierarchical clustering grouped the cultivars into two main groups (Figure 4). The grouping, however, only roughly correlated with genetic relationship, contrary to the previous observation [9]. Allurel and Natyra, which share a parent, formed neighboring clusters. Topaz and Ladina (parent and offspring) grouped together in peel extracts’ analyses, but only when the dominating sugars were not considered. Elstar and Zari (parent and offspring), on the other hand, did not even belong to the same main cluster.

Since eight of the investigated cultivars were scab and mildew resistant and another two scab resistant (Table 1) we also reassessed the predictive potential of the fruit metabolome for this purpose. A positive outcome of such an analysis would be very interesting for breeding purposes and was postulated in a recent publication where a high fruit content in polyphenolic compounds was discussed as potentially effective against pests [15]. In our analysis, however, we failed to detect a connection between the fruit metabolome and SMR. Resistant cultivars were equally distributed between the two main cluster groups and no grouping within the subgroups was discernible that could be related to scab or mildew resistance.

In summary, NMR-based metabolomics analyses of apple varieties can provide a simple non-targeted and straightforward method to give a comprehensive view on the metabolomics variety of all medium to highly concentrated analytes. Cultivar-specific differences in the profiles of nonvolatile metabolites allowed even within a set of rather similar cultivars a correct and easy classification. We are convinced that this approach will prove its high potential for identification and validation in view of food authenticity applications.

## 4. Materials and Methods

### 4.1. Plant Material and Sampling

Apples were cultivated in a pesticide-reduced system at the Competence Center of DLR Rheinpfalz, Klein-Altendorf, Germany. In total, 14 cultivars were investigated of which 13 (“Allurel”, “Crimson Crisp”, “Galiwa”, “Gemini”, “Isaaq”, “Ladina”, “Lubera”, “Natyra”, “Pinova Evelina”, “PRI010”, “PRI037”, “Roter Topaz”, and “Zari” ) were newly bred or brought onto the market in recent years. “Elstar van der Zalm” served as a reference cultivar. Fruits were harvested at commercial maturity in August or September 2014, randomized and stored at 2–3 °C until transport to the lab in Karlsruhe. Apples were stored at 1 °C and a humidity of 95% until samples were probed in October 2014.

For each cultivar, 10 samples were collected, combining material from five fruits each. Peel and pulp samples were taken separately. Peel was sampled by cutting two longitudinal thin strips from opposite sides with a ceramic scalpel. After immediate freezing in liquid nitrogen, the peel was ground and transferred to 20 mL screw-cap glasses. After removing the surrounding peel, fruit pulp was sampled at the same spot as the peel by cutting off small pieces with a ceramic knife. Pulp samples were further treated as the peel samples. The frozen material was lyophilised for 72–84 h (Alpha 2–4 LSC, Christ, Osterode, Germany). As the dried peel samples of the different cultivars had a different firmness, the grinding procedure was done in a cultivar-specific way in order to obtain a comparable grinding result. Usually, peel samples were first ground with a knife mill (A11 basic, IKA GmbH & Co., KG, Staufen, Germany) for 1 min (if necessary) and finally milled with a ball mill (MM200, Retsch GmbH, Haan, Germany) for 2–4 min at 25 Hz. In case of pulp samples, milling with the ball mill for 1 min at 25 Hz was always sufficient. All samples were stored until further analysis in 2 mL Eppendorf tubes at −80 °C.

### 4.2. Sample Preparation

Samples were extracted with 10 mg/200 µL buffer. Acetate-*d*_4_ buffer (200 mM acetate-*d*_4_, pH 4.08, 1 mM TSP, 0.2 mM EDTA, 9:1 H_2_O/D_2_O) was used for peel, and phosphate buffer (200 mM, pH 3.04, 1 mM TSP, 0.2 mM EDTA, 9:1 H_2_O/D_2_O) for pulp. Samples were resuspended and then incubated at room temperature for 20 min. Thereafter, the samples were centrifuged at 11,000 *g* for 30 min (apple pulp) and 45 min (apple peel). Six hundred microliters of the supernatant were transferred to 5 mm NMR tubes (Duran group) and were measured within 24 h. Quality control samples consisted of samples pooled from all cultivars.

### 4.3. ^1^H-NMR Spectroscopy

An ^1^H-NMR (NOESYGPPR1D) and a 2D J resolved spectrum was recorded for each sample at 300 K on a 600 MHz Avance II spectrometer (Bruker Biospin, Rheinstetten, Germany) using a double resonance 5 mm BBI probe with actively shielded z-gradients. Data acquisition and processing were carried out with Topspin 3.2. The water signal was suppressed using a presaturation pulse with a bandwidth of 25 Hz. Spectra were acquired with 32 scans, 96 k data points, a spectral width of 30 ppm and a relaxation delay of 10 s. Spectra were automatically processed, phase- and baseline-corrected using an exponential window function with a line-broadening factor of 0.3 Hz. They were calibrated setting the TSP signal to 0 ppm. Quality control samples were regularly measured (1 quality control sample per 10 samples) to control the reproducibility of the measurements with time. 

### 4.4. Data Processing of the NMR Spectra and Multivariate Pattern Recognition

NMR spectra data were normalized to total intensity between 0.6 and 9.7 ppm and integrated following a variable sized bucketing pattern in AMIX 3.9.9 (Bruker Biospin, Rheinstetten, Germany), excluding the regions corresponding to noise and water/HOD (δ 4.73–4.83 ppm). Chemical shift assignment was achieved by comparison with database entries (FOODB [18], HMDB [19]), literature, or through spiking with pure substances (Figure S3). Bucket tables were imported in JMP genomics version 12.2.0 (SAS, Cary, NC, USA) for multivariate analysis. Data were scaled to unit variance to take into account also small signals. Principal components analysis (PCA) was used as an unsupervised pattern recognition method. In addition, the data were analyzed by hierarchical clustering algorithms (complete linkage and Pearson’s correlation distance methods).

## 5. Conclusions

We could show that for a set of apple cultivars correct classification can be achieved by principle component analysis of peel or pulp extracts. Further in-depth analysis failed to correlate the extracted metabolites with the genetic relationship between cultivars, contrary to previous studies. Likewise, we had to reject the hypothesis that fruit metabolites’ analyses can yield easy criteria to identify scab or mildew resistance, at least for our selection of cultivars. We therefore conclude that the untargeted NMR analysis of peel or pulp extracts presents an efficient tool to differentiate between closely related species and/or for quality control, however it may not be *a priori* suitable to be used in more advanced applications.

## Figures and Tables

**Table 1:** Apple cultivars used in this study.

Cultivar	Characteristics
Galiwa	Scab mildew resistant (SMR)
Pinova Evelina	Established cultivar, colour mutant
Elstar v. d. Zalm	Established cultivar
Ladina	SMR
Red Topaz	SMR
PRI 037	SMR, bred in Wageningen
Gemini	Scab resistant, from Italy
Zari	from Belgium
PRI 010	SMR
Crimson Crisp	SMR, established cultivar from USA
Isaaq	Scab resistant, from Italy
Allurel	SMR
Natyra	SMR
Lubera	red-fleshed

## Supplementary Materials

The following are available online at www.mdpi.com/2218-1989/6/3/29/s1, Figure S1: pH shifts observed for peel extracts exemplified for four different cultivars; Figure S2: sample variability within pulp extracts; Figure S3: Spiking with pure substances to identify polyphenolic compounds; Figure S4: Sample variability between the cultivars; Figure S5: PCA two-dimensional views; Figure S6: Reproducibility of the method; Table S1: List of identified compounds.
