# Supplementary material for: Untargeted NMR Spectroscopic Analysis of the Metabolic Variety of New Apple Cultivars
Source: Metabolites. 2016 Sep 19;6(3):29. doi: 10.3390/metabo6030029 (PMC5041128; doi:10.3390/metabo6030029)
Supplement: Supplementary file 1 [file metabolites-06-00029-s001.pdf]

Supplementary Materials: Untargeted NMR Spectroscopic Analysis of the Metabolic Variety of New Apple Cultivars

Philipp Eisenmann, Mona Ehlers, Christoph Weinert, Pavleta Tzvetkova, Mara Silber, Manuela J. Rist, Burkhard Luy and Claudia Muhle-Goll

Table S1. NMR signals used for analysis.

ppm	Compound
0.91, 1.01	Isoleucine
0.98, 1.04	Valine
1.32	Lactate
1.47	Alanine
1.88, 1.98	Quinic acid
2.05	Acetate
2.64, 2.82, 4.39	Malic acid
2.68, 2.88, 2.89	Citrate
3.19	Choline
3.21, 3.31, 3.51, 3.92, 4.56, 4.58, 4.64, 4.19	Xylose
3.22, 3.24, 3.40, 3.45, 3.52, 3.73, 5.22	Glucose
3.25, 3.27, 3.62	myo-Inositol
3.20, 3.67, 3.75, 3.77, 4.05, 4.21, 5.40	Sucrose
3.60, 3.65, 3.70, 4.00, 4.10	Fructose
4.33, 4.98, 6.09	Epicatechin
4.55	Tartrate
4.57, 4.59, 5.26	Galactose
5.49	Arabinose
1.26	Rhamnitol
1.45, 2.71, 2.74	Citramalic acid
2.37	Pyruvate
3.34	Betaine
6.38, 7.19, 7.65	Chlorogenic acid
6.11, 6.25, 6.83, 7.14	Phloricine
Overlap:	
3.48, 3.88	Glucose, Sucros
3.56, 3.81	Glucose, Fructose, Sucrose
4.03	Sucrose, Fructose

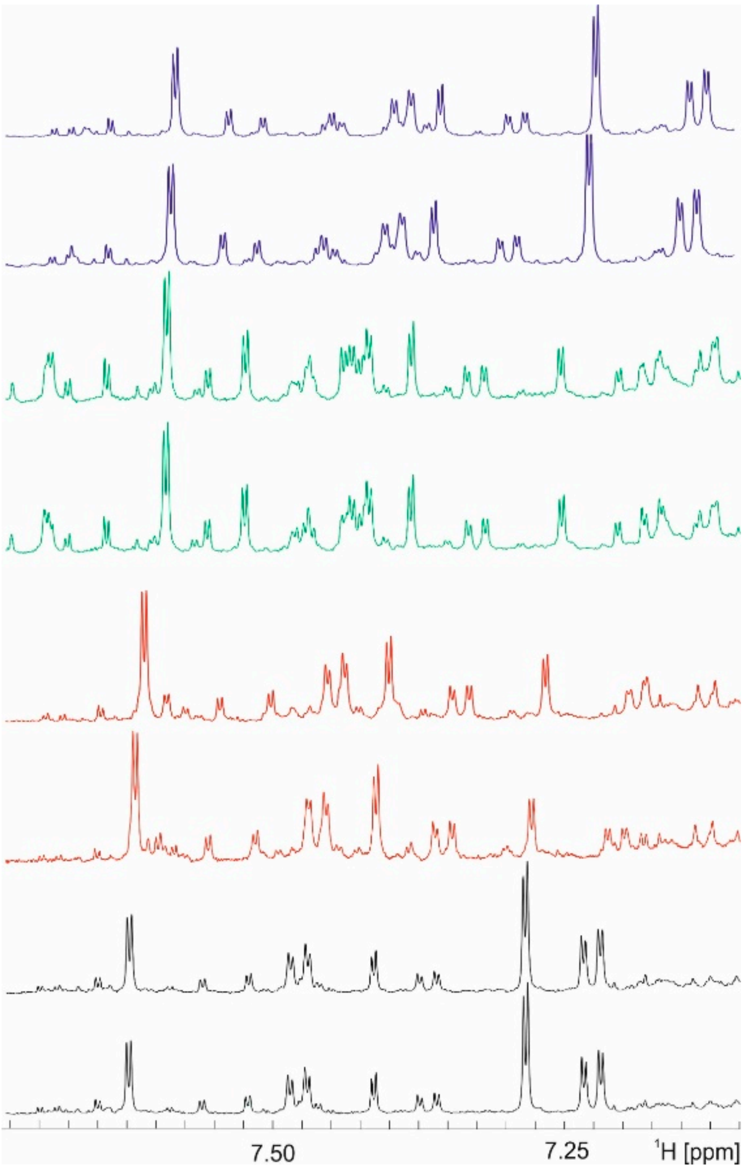

Figure S1. pH-Shifts observed for peel extracts exemplified for four different cultivars. For the sake of clarity only two spectra, respectively, are shown. Black: Red Topaz, red: Galiwa, green: Isaaq, blue: Ladina.

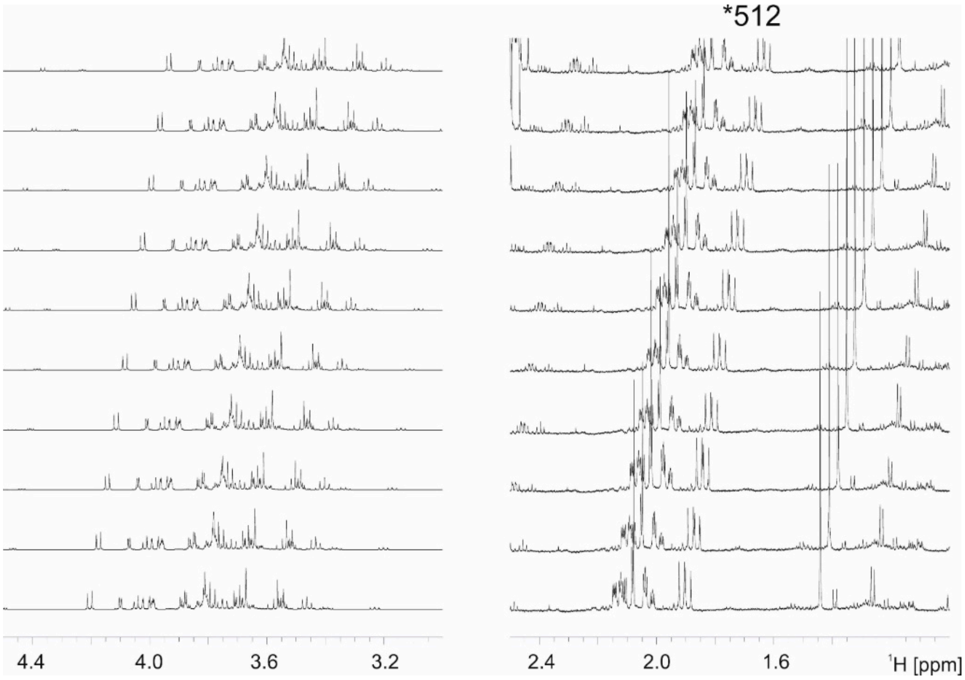

Figure S2. Sample variability within pulp extracts (here: Galiwa). The sugar (**left**) and aliphatic (**right**, multiplied by a factor of 512) regions of the spectra are shown.

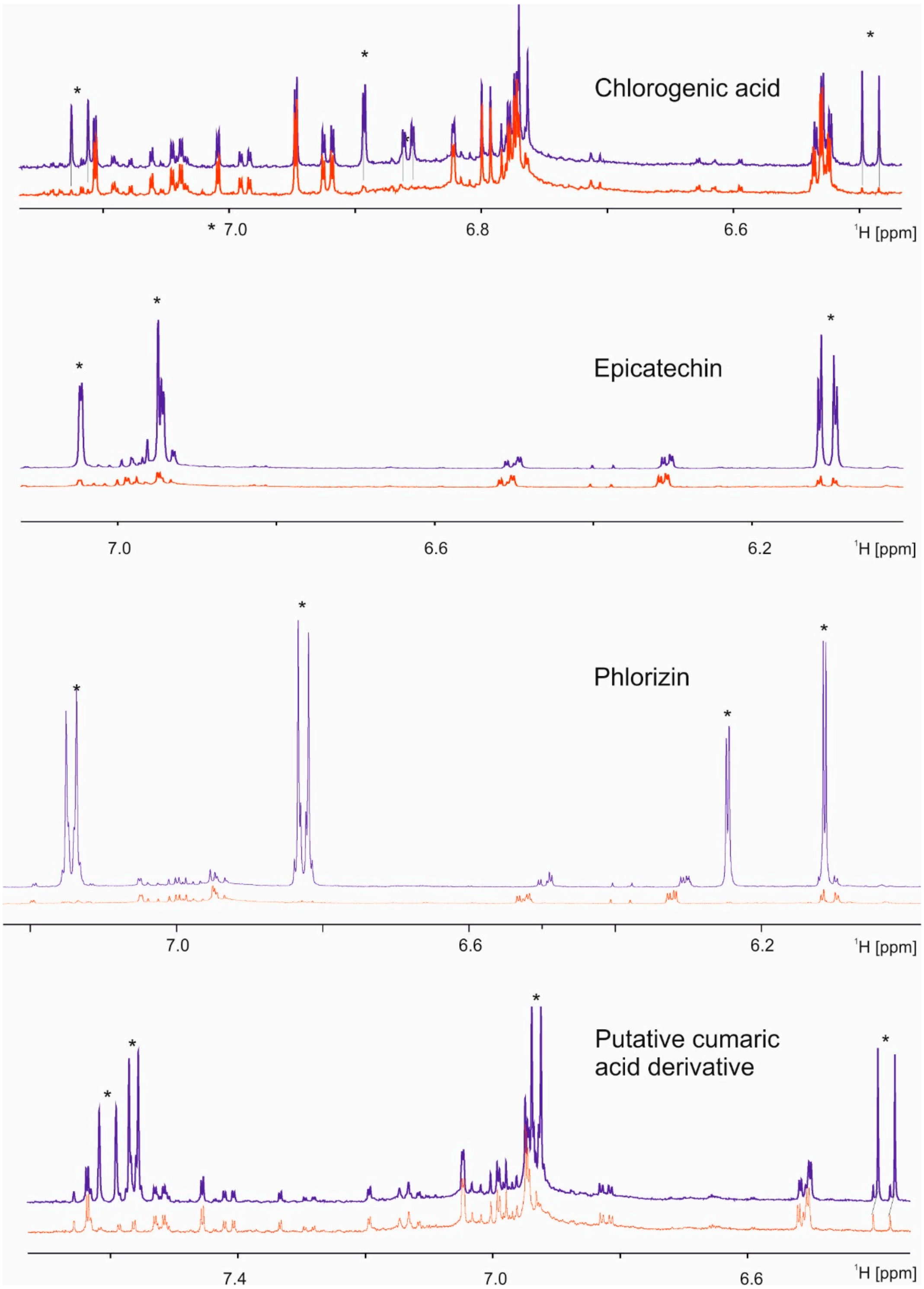

Figure S3. Spiking with pure substances to identify polyphenolic compounds. * denotes the resonances of the spiked substances.

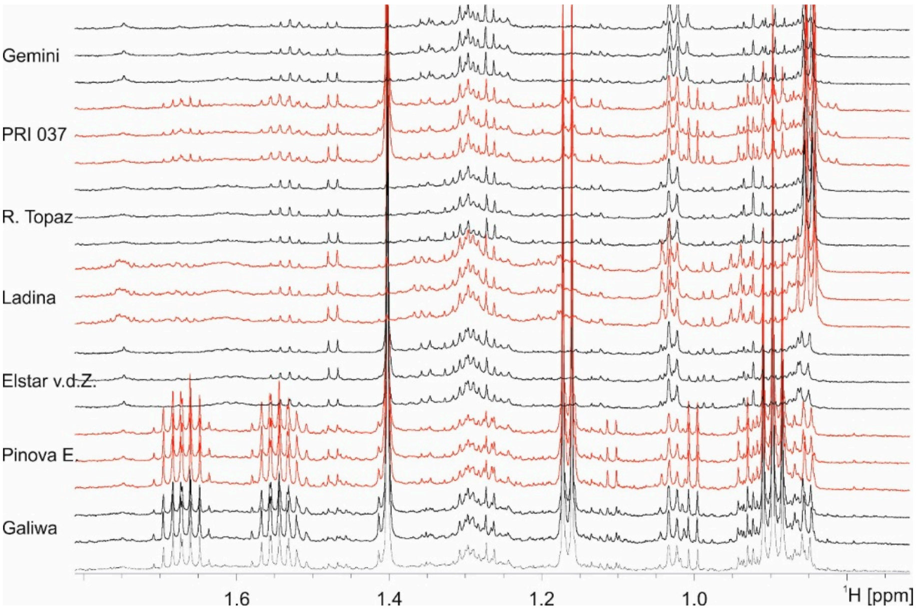

Figure S4. Sample variability between the cultivars.

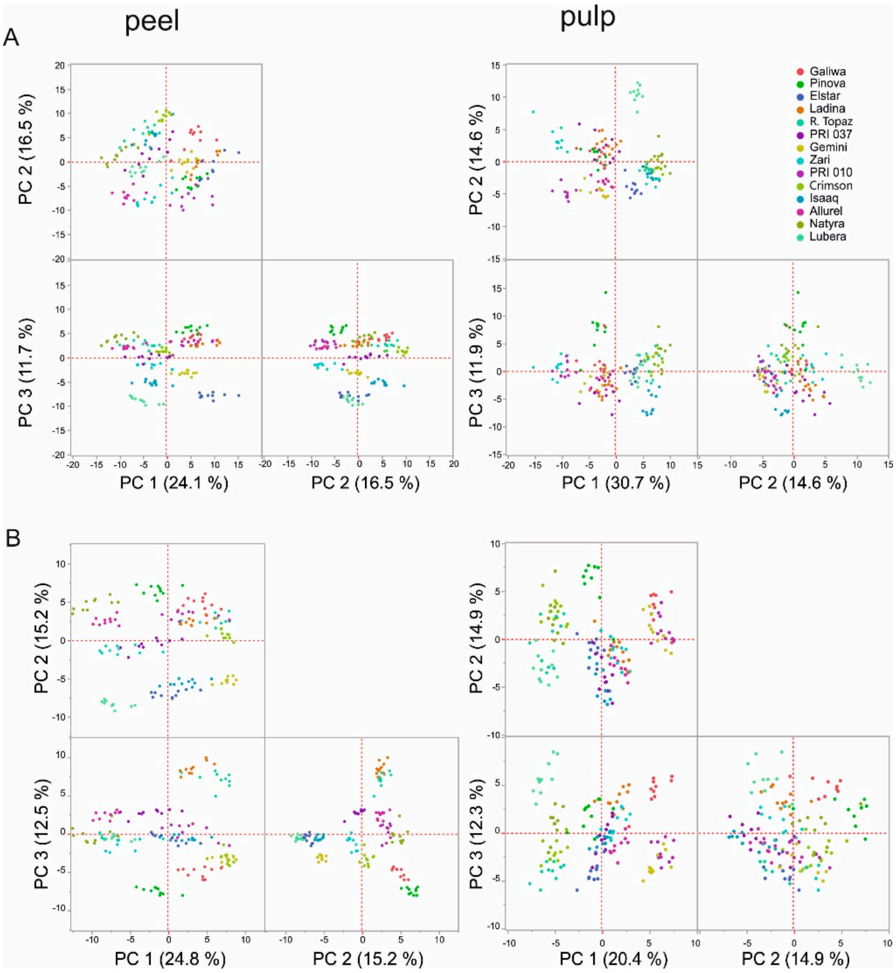

Figure S5. PCA two-dimensional views. (**A**) All buckets were used for the analysis. (**B**) Main sugar resonances were omitted from the analysis.

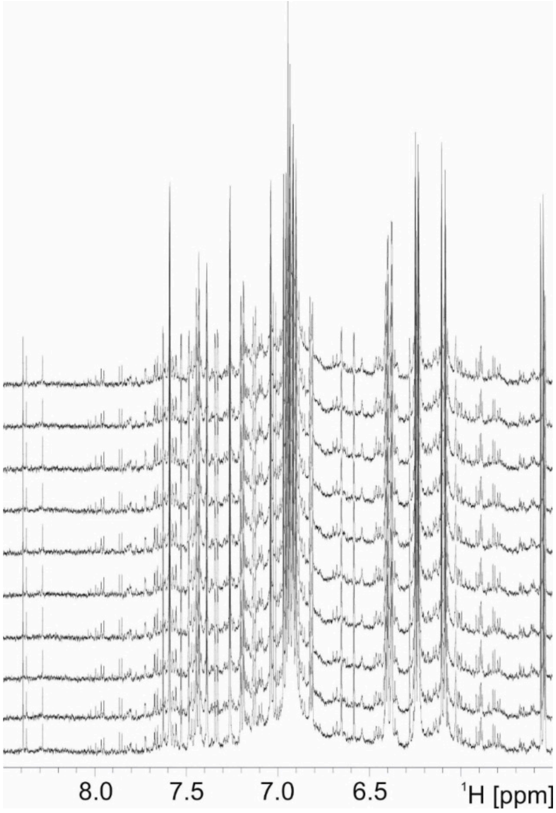

Figure S6. Reproducibility of the method. The aromatic region of ten different quality control samples is shown.
